# Acute Schistosomiasis in Brazilian Traveler: The Importance of Tourism in The Epidemiology of Neglected Parasitic Diseases

**DOI:** 10.1155/2012/650929

**Published:** 2012-07-16

**Authors:** Diego Averaldo Guiguet Leal, Regina Maura Bueno Franco, Maria Francisca Neves, Luciana Franceschi Simões, Letícia Aparecida Duart Bastos, Silmara Marques Allegretti, Eliana Maria Zanotti-Magalhães, Luiz Augusto Magalhães

**Affiliations:** Departamento de Biologia Animal, Instituto de Biologia, Universidade Estadual de Campinas, Rua Monteiro Lobato No. 255, P.O. Box 6109, 13083-970 Campinas, SP, Brazil

## Abstract

Parasitic infectious diseases acquired in tourist areas may pose a challenge to physicians and to travel medicine practitioners. Acute schistosomiasis may be seen in returning travelers and migrants after primary infection. This form of schistosomiasis is frequently misdiagnosed due to its temporal delay and its nonspecific presentation and might occur even in countries where the disease is endemic, such as in Brazil. The patient developed the acute phase of schistosomiasis with severe clinical manifestations. The quantitative analysis revealed the presence of 240 eggs per gram of stool. The treatment was administered with oxamniquine, and the control of cure of the patient was monitored and was favorable. The present paper aims to emphasize the importance of a detailed clinical history including information regarding travel history.

## 1. Introduction

Human schistosomiasis is an important intravascular infection caused by parasitic trematode helminths and still represents an important public health problem in different parts of the world. Approximately 207 million people are infected worldwide and the disability-adjusted life years (DALYs) associated to schistosomiasis is estimated in 1.5 to 4.5 million per year [1, 2].

*Schistosoma* *mansoni*, which inhabits the host mesenteric portal system, is the only species found in Brazil. In present times, 25 million people are living in endemic areas in Brazil, and 4 to 6 million are infected [3, 4]; however, there are areas in the country where the foci of the disease remain unknown. 

In a chronic phase, the severity of the disease and its pathology are closely related to the presence of *Schistosoma* *mansoni* eggs that become trapped in tissues, especially in liver, and can lead to periportal fibrosis and hepatosplenic schistosomiasis [1]. Otherwise, the acute phase of schistosomiasis may have a very sudden onset with clinical signs and symptoms occurring within a few weeks to months after primary infection [5, 6].

This form of schistosomiasis is characterized by a temporal delay and nonspecific presentation; for this reason, it is the phase of the disease most likely to be misdiagnosed by physicians and by travel medicine practitioners [7]. In nonimmune individuals or those who have never had primary infection, such as travelers and migrants, the acute phase may be diagnosed as another ailment or may not be part of the diagnosis hypothesis even in countries like Brazil, where it is endemic in some areas. The present paper intends to emphasize the importance of a detailed clinical history, including information regarding travel history. 

## 2. Case Presentation

**Figure 1:**
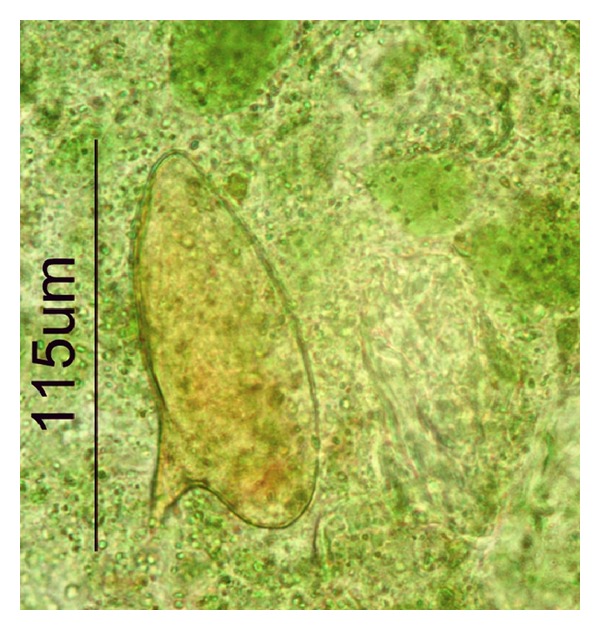
*Schistosoma mansoni* eggs detected by Kato-Katz method from patient's stool.

A male 32-year-old patient, who lives in Campinas, São Paulo, Brazil, was admitted to a private hospital in his city. He had traveled on vacation to the state of Bahia, (Northeastern Brazil) Barra Grande city, on New Year's Eve 2008. After approximately one month of return, the patient reported a sudden onset of symptoms and the clinical pattern at admission to hospital included fever (39°C), fatigue, malaise, persistent and nonproductive cough, and diarrhea. The patient was examined by a physician and, after a chest radiography, the putative diagnosis at that time was pneumonia and antibiotic treatment was started at home. One week after the treatment the patient remained with continuous lassitude and diarrhea with some blood and persistent cough and was examined by other physicians that reported the same symptoms. Routine laboratory examinations were performed: complete blood count (*n* = 3) in alternate days for one week and one stool parasitological examination for screening intestinal parasites. The only alteration detected in the blood count was eosinophilia, which showed a gradual increase once every two days: 816, 1,232, and 1,408 cells/*μ*L percentage corresponding to 8.0%, 14.0%, and 16.0%, respectively, of total leukocytes (normal range 0–7.0% or ≤500 cells/*μ*L). The parasitological investigation—fresh stool examination—revealed trophozoites of *Entamoeba* *histolytica/Entamoeba* *dispar*. The patient was treated with secnidazole, 2,000 mg at that moment and 2,000 mg after 14 days. About two weeks after the treatment, the patient remained with the same signs and symptoms exhibited before the diagnosis of pneumonia and amebiasis and his clinical pattern worsened: very strong cough, progressing to dyspnea, generalized rash, and marked weight loss (about 8 pounds since the onset of symptoms). One week later, a new routine laboratory investigation showed leukocytosis, presence of atypical lymphocytes, normocytic-normochromic anemia, and high eosinophilia: 4,560 cells/*μ*L—40.0% of total leukocytes. The patient was hospitalized and submitted to infectious diseases screening, which revealed positive serology to Epstein-Barr virus (EBV), cytomegalovirus (CMV) and hepatitis A virus (IgG-ELISA) for all viruses, data consistent with past infections. Serology for hepatitis B and C viruses, HIV types I and II, *Toxoplasma gondii,* and *Toxocara* *canis* was all negative. The stool parasitological examination evidenced the presence of *Entamoeba coli *and *Endolimax* *nana,* and hemocultures were negative. After the inconclusive diagnosis and a suspicion of infection with helminths, the patient was referred to the Department of Animal Biology, Sector of Parasitology, of Biology Institute in State University of Campinas. The patient showed all the exams and was interviewed about his signs and symptoms and about information regarding travel history. Important epidemiological data and clinical pattern were obtained from that holiday: the patient had gone through a small dam to get to a beach (Taipus de Fora) rarely explored by tourists and often frequented by autochthonous population; the exposure time was approximately five minutes. The patient also recalled that he had a localized pruritic rash in both legs—cercarial dermatitis—after crossing the weir. Prior parasitological stool testing was negative on prior days until the one done at 70 days. The Kato-Katz method [8] as well as the flotation procedure of Willis and spontaneous sedimentation method was applied in the Sector Parasitology in the State University of Campinas to search for helminthes. The coproparasitological analysis showed many Charcot Leyden crystals, and the Kato-Katz method showed the presence of *Schistosoma mansoni *eggs with typical lateral spine (Figure 1). The quantitative analysis revealed the presence of 240 eggs per gram of stool. The patient was treated with Oxamniquine (Mansil)—single dose of four tablets of 250 mg, according to the weight of the patient. The control of cure was performed for four successive months after the treatment using the Kato-Katz method to search for eggs. All stool samples were exposed to light for about 2 hours to stimulate eclosion of the miracidia. The monitoring of the control of cure to search for eggs or larvae was all negative for *Schistosoma* *mansoni,* and the result of the treatment was favorable.

## 3. Discussion 

Exposure to schistosomiasis may pose a health risk to travelers that visit certain endemic areas. It is a well-known fact that this disease represents an important public health problem since its transmission is strongly associated with deficiencies in sanitary sewage treatment. Presentation of signs and symptoms such as high fever, nonproductive cough, diarrhea, fatigue, and malaise similar to that of many infectious diseases and will not raise a suspicion of acute schistosomiasis. A straightforward diagnosis might be done when considered together, the clinical pattern of the patients within their specific travel history, associated with specific activities, that is, exposure to cercaria in contaminated water. When a noninfectious cause of eosinophilia is excluded from returning travelers, it might lead the physician or other travel medical practitioner to search for infectious diseases, considering that, by far, the infectious diseases strongest associated with eosinophilia are helminthiasis [9]. 

Schistosomiasis is an important cause of eosinophilia among travelers returning from tropical or subtropical countries [9, 10]. The linkage between schistosomiasis and tourism is growing, and many outbreaks of acute schistosomiasis have been documented especially among those individuals who practice “thrill sports” like diving, water skiing, and rafting [11–13]. In endemic regions the acquisition of the disease is also associated with recreational activities such as bathing and swimming in freshwater bodies of water. 

In the present case, *Schistosoma mansoni* was the confirmed species. The diagnosis was finally established by a routine stool examination (Kato-Katz method). The parasitological examination and the demonstration of *Schistosoma mansoni* eggs are considered the most reliable method for diagnosis of schistosomiasis. However, in heavy infections related with the acute phase of the disease it is often difficult to detect *Schistosoma* eggs and serological tests are frequently negative initially [7, 14]. Furthermore, the appropriate parasitological technique must be used when there is a suspicion of schistosomiasis and the stool analyzed at least four weeks after human contact with cercaria in contaminated bodies of water. Mucosal biopsy was not considered for the diagnosis of schistosomiasis, probably because at that time the suspicion of infection by this trematode was not raised, and must be used with caution. In the present case, an expressive concentration of Charcot-Leyden crystals was detected; this fact can also be an indication of intestinal parasitism by helminths, since this is the result of breakage of eosinophils that are attracted to or produced in larger amounts in an attempt to contain helminthes infections [15]. Even brief contact with bodies of water contaminated with cercaria of *Schistosoma mansoni* (less than five minutes) resulted in infection rates ranging from 30,0% to 77,0% of the portion of exposed tourists who became infected [11, 13, 16, 17]. The short period of exposure to contaminated water and *Schistosoma mansoni* infection was also evidenced in the present case since the patient entered only once in the contaminated water and spent approximately five minutes there. Actually, praziquantel is the drug of choice for the treatment of infection of different species of *Schistosoma* [18]; in the present study, the administration of Oxamniquine was favorable considering its curative activity when following up the control of the cure of the patient. This drug may be an alternative treatment option against *Schistosoma mansoni;* however, it is very difficult to obtain at present because there is low demand for it [14]. 

## 4. Conclusions

This paper highlights the importance of a detailed clinical history including brief water exposures and repeated stool examinations among travelers presenting with unusual clinical presentations and eosinophilia, in the absence of serology or other diagnostic options to identify acute schistosomiasis, especially after primary infection.
